# Drought reduces water uptake in beech from the drying topsoil, but no compensatory uptake occurs from deeper soil layers

**DOI:** 10.1111/nph.17767

**Published:** 2021-10-15

**Authors:** Arthur Gessler, Lukas Bächli, Elham Rouholahnejad Freund, Kerstin Treydte, Marcus Schaub, Matthias Haeni, Markus Weiler, Stefan Seeger, John Marshall, Christian Hug, Roman Zweifel, Frank Hagedorn, Andreas Rigling, Matthias Saurer, Katrin Meusburger

**Affiliations:** ^1^ Research Unit Forest Dynamics Swiss Federal Research Institute for Forest, Snow and Landscape Research WSL 8903 Birmensdorf Switzerland; ^2^ Institute of Terrestrial Ecosystems ETH Zurich 8092 Zurich Switzerland; ^3^ Hydrology, Faculty of Environment and Natural Resources University of Freiburg 79098 Freiburg Germany; ^4^ Department of Forest Ecology and Management Swedish University of Agricultural Sciences Umeå 90283 Sweden; ^5^ Research Unit Forest Soils and Biogeochemistry Swiss Federal Research Institute for Forest, Snow and Landscape Research WSL 8903 Birmensdorf Switzerland

**Keywords:** Bayesian isotope mixing model, drought, drought release, European beech (*Fagus sylvatica*), oxygen isotopes, soil water, tree water use, xylem water

## Abstract

- The intensity and frequency of droughts events are projected to increase in future with expected adverse effects for forests. Thus, information on the dynamics of tree water uptake from different soil layers during and after drought is crucial.
- We applied an *in situ* water isotopologue monitoring system to determine the oxygen isotope composition in soil and xylem water of European beech with a 2‐h resolution together with measurements of soil water content, transpiration and tree water deficit. Using a Bayesian isotope mixing model, we inferred the relative and absolute contribution of water from four different soil layers to tree water use.
- Beech took up more than 50% of its water from the uppermost 5 cm soil layer at the beginning of the 2018 drought, but then reduced absolute water uptake from the drying topsoil by 84%. The trees were not able to quantitatively compensate for restricted topsoil water availability by additional uptake from deeper soil layers, which is related to the fine root depth distribution. Absolute water uptake from the topsoil was restored to pre‐drought levels within 3 wk after rewetting.
- These uptake patterns help to explain both the drought sensitivity of beech and its high recovery potential after drought release.

## Introduction

Water is one of the central limiting resources for plant growth and ecosystem functioning (Churkina & Running, 1998). The ongoing climate change with projected increases in the frequency and intensity of drought events in future will further increase the importance of water availability for terrestrial ecosystems. Intense drought events not only strongly reduce ecosystems’ primary productivity (Ciais *et al*., 2005) but also lead to large‐scale mortality events, especially in trees and forest ecosystems (Allen *et al*., 2010, 2015). The main water resource for trees is soil water, the amount and availability of which shows strong temporal variations due to the variability of precipitation input into the soil (Porporato *et al*., 2004). Trees take up the water from different layers of the soil via their fine roots and partially via mycorrhizal hyphen (Allen, 2007) and thus, a match between how the available water is vertically distributed and where the roots are located is important for tree water use. Trees may adjust their rooting system to changing soil moisture conditions (Poorter *et al*., 2012), but it remains uncertain how tree water use copes with extreme drought events during which the upper soil layers, where fine roots are most abundant, dry out quickly.

Numerous studies have assessed the origin of water taken up by a tree (see review by Sprenger *et al*., 2016). This is mainly done by either labelling particular water sources with water that is artificially enriched or depleted in the heavier oxygen isotope (^18^O) or hydrogen isotope (^2^H) or by taking advantage of the natural isotopic differences of different water sources. Since root water uptake is generally a nonfractionating process (Dawson & Ehleringer, 1991), proportional contributions of different sources to the water used by trees can be quantified by comparing the isotopic signature of these potential water sources with xylem or transpiration water, and by applying isotope mixing models (Parnell *et al*., 2013). Research has been especially focusing on the vertical distribution of water uptake from the soil (Plamboeck *et al*., 1999; Stahl *et al*., 2013), the uptake of ground vs soil water (Costelloe *et al*., 2008; Barbeta & Peñuelas, 2017) or of soil vs stream water (Dawson & Ehleringer, 1991; Bowling *et al*., 2017). Due to time‐consuming analysis with traditional methods, most of these studies, however, only provide a temporal resolution of weeks or lower (e.g. Brandes *et al*., 2007; Brinkmann *et al*., 2019) and might thus not be able to capture short‐term dynamics of root water uptake during particular drought or precipitation events within a growing season.

With five to nine sampling times per year over three growing seasons, Brinkmann *et al*. (2019) showed that four common European tree species (*Fagus* *sylvatica*, *Acer pseudoplatanus, Fraxinus excelsior and Picea abies*) used water mostly from shallow soil layers when water availability was high. Three of the species (*F. excelsior*, *F. sylvatica* and *A. pseudoplatanus*) were, however, able to shift proportional water uptake to deeper soil layers, when water availability decreased in the topsoil. Comparable patterns of an increased relative contribution of deeper soil water to water uptake of beech with increasing topsoil dryness were observed by Seeger & Weiler (2021). Likewise, isotopic analyses of soil and xylem water during a summer drought indicated that *Quercus petraea* and *Pinus sylvestris* both took up water from near surface in monoculture, but oak was able to exploit deeper water resources in mixture (Bello *et al*., 2019). Soil and stem oxygen and hydrogen isotope sampling with moderately high resolution (2–4 d wk^−1^) in a subtropical conifer plantation revealed that tree water sources shifted to deeper soil layers with a seasonally progressing drought (Yang *et al*., 2015). However, these results contrast with the findings of Lüttschwager & Jochheim (2020). They applied a mechanistic ecosystem model revealing that in the drought year 2003 beech trees not only reduced total water use but also decreased the relative water uptake from deeper soil layers.

Sporadic observations of water isotopes have provided valuable insights into how isotopic signatures change through the vadose zone and plant xylem. However, it is unclear how increased sampling frequencies in space and especially in time would affect estimates of the potential sources of root water uptake and their shifts under variable water availability.

Only recently the application of laser isotope spectrometers allowed very high (up to hourly) temporal resolution assessments of water isotope composition in the soil (Gangi *et al*., 2015; Volkmann *et al*., 2016a), in the tree xylem (Volkmann *et al*., 2016b; Marshall *et al*., 2020; Seeger & Weiler, 2021) or in equilibrium plant transpiration (Volkmann *et al*., 2016a; Lanning *et al*., 2020). Such high‐frequency information on the vertical distribution of the soil water isotopic composition and on the water transported within the tree gives insights not only in the depth distribution of soil water uptake of trees but also in its short‐term dynamics – especially during drought and soil rewetting. Volkmann *et al*. (2016a) showed species‐specific differences in water uptake dynamics of tree seedlings during and after a mild drought: While sessile oak adjusted root water uptake to vertical water availability patterns under drought, the readjustment of uptake towards a rewetted topsoil was delayed. By contrast, European beech readily utilized water from all soil depths independent of water depletion during the mild drought, enabling faster uptake of rainwater after the drought was relieved. While this study provided information on water uptake dynamics in close to hourly time resolution, it did not assess longer‐term (i.e. seasonal) variation of root water uptake. Such longer‐term information (still with high temporal resolution) is however, needed to understand seasonal water use and potential acclimations to drought periods, as well as variable recovery afterwards.

Even within a given soil depth, different water pools can exist, which might or might not be available for plant water uptake. The ‘two water worlds’ hypothesis assumes that mobile water in the soil drains vertically and contributes to groundwater recharge, whereas more tightly bound water is available for plants (McDonnell, 2014). This hypothesis is, however, questioned by well‐established physiological and physical mechanisms (see discussion by Bowling *et al*., 2017). Only recently it has been assumed that observed mismatches in the deuterium isotope signatures between potential source water and water extracted from the plant xylem could be a result of cryogenic extraction artefacts caused by an exchange between organically bound hydrogen and water (Chen *et al*., 2020). We need, however, also to acknowledge that the traditional methods used for extracting soil water (e.g. cryogenic distillation (Ehleringer *et al*., 2000)) to measure its oxygen and hydrogen isotope composition obtain bulk soil water with an isotopic composition that might not be representative for the water taken up by trees (Brooks *et al*., 2010). It is thus important to assess if source water isotopic signatures depend on the extraction method since such a bias would strongly affect the estimates of the origin of water and its dynamics.

Our aim was to assess the source of water taken up by mature European beech (*F. sylvatica* L.) trees over the growing season of the extremely hot and dry year 2018 (Schuldt *et al*., 2020). Beech is an ecologically and economically important tree species in Europe (Hanewinkel *et al*., 2013) but will be likely impaired in its functioning by increasing drought under climate change (Gessler *et al*., 2007). We here applied the *in situ* water isotopologue monitoring system (Volkmann & Weiler, 2014) to determine the oxygen isotope composition in the soil (Volkmann *et al*., 2016a) and beech xylem water (Volkmann *et al*., 2016b) in a mixed beech forest at close to 2‐h resolution. Moreover, we compared the oxygen isotopic composition of water vapour in equilibrium with soil and xylem water obtained from the *in situ* method with water samples that were cryogenically distilled and measured with isotope ratio mass spectrometry (IRMS) to verify potential extraction method artefacts relevant for tracking the origin of water taken up by trees with oxygen isotopes.

We hypothesized that (hypothesis 1) beech would be able to continuously access more water from deeper soil layers as the topsoil dried out during the extreme drought in 2018, at least partially compensating for the declining water uptake from surface soils. We further assumed that (hypothesis 2) beech would quickly recover its water uptake from the topsoil once it is rewetted.

## Materials and Methods

### Field site and meteorological, soil and tree physiological measurements

The study was carried out in a mixed beech forest composed of Norway spruce, sycamore maple, Norway maple in addition to beech. The stand is flat and located close to the Swiss Federal Research Institute WSL in Birmensdorf, Switzerland (47.363°N, 8.454°E; 550 m above sea level (asl)). The soil is characterized as a loamy Pararendzina formed from glacial moraines showing signs (hydromorphic oxidation zone) of temporary waterlogging below 50 cm soil depth. Long‐term mean annual and mean summer (June, July, August (JJA)) air temperature amount to 9.5 and 17.7°C, respectively and the average yearly and average summer precipitation are 1124 and 377 mm (MeteoSwiss, Station Zurich‐Fluntern).

The experiment was carried out from 10 May to 16 September 2018. Air temperature and relative air humidity (Rotronic, HC2S3, Temperature and Relative Humidity Probe; Rotronic GmbH, Esslingen, Germany) and precipitation (ARG100, Tipping Bucket Raingauge, 0.2 mm/tip; Campbell Scientific Ltd, Bremen, Germany) with a resolution of 10 min were determined in close vicinity of the forest stand (200 m distance) in an open area. Water pressure deficit of the air (VPD) was calculated from air temperature and relative air humidity according to Jones (1992). Volumetric soil water content (VWC) and soil temperature were measured in the forest stand in one of the two soil profiles used for isotope probing (see later and Supporting Information Table S1). ECH2O 5 TE sensors (Meter Environment, Munich, Germany), that were used to measure VWC and soil temperature, were installed in 5, 15, 30 and 45 cm depth and data was stored as 10 min average with a CR1000 logger (Campbell Scientific, Bremen, Germany). Soil matric potential (Ψ_soil_) and soil temperature were further measured in another soil profile in two depths (5 cm and 30 cm) at hourly intervals with MPS‐2 sensors (Decagon Devices, Pullman, WA, USA). The soil matric potential measurements were temperature corrected to 22°C according to the procedure of Walthert & Schleppi (2018).

We selected three dominant beech trees (Tree nos 47, 49, and 50) with diameters at breast height (dbh) between 31.5 and 47.2 cm and a height of *c*. 20 m in close vicinity to the soil profiles. These trees were equipped with constant heating xylem flow sensors (Granier *et al*., 1996) connected to a control and logger unit (Xylemfluss‐Mess‐System M1; UP GmbH, Ibenbühren, Germany). The two needles of the sensors were drilled 5 cm into the sapwood and insulated from direct sunlight. Data were logged as flux densities (FD values: l cm^−2^ s^−1^) based on the sapwood area (SA) with 10 min temporal resolution. SA of a given tree was calculated from the relationship between dbh and SA (cm^2^) determined for 25 beech trees according to Keitel *et al*. (2003). Transpiration per tree (*T*_t_ in l s^−1^) was calculated as FD × SA. For daily sums of ground area‐based transpiration (l m^−2^ d^−1^ = mm d^−1^), daily *T*_t_ was divided by the crown projection area of the trees (between 41.4 and 49.4 m^2^). In addition, stem temperature was determined in the sapwood of one tree. Stem radius changes were measured with point dendrometers at breast height (ZN11‐T‐WP; Natkon, Oetwil am See, Switzerland) in the same three beech trees. The dendrometers, including cables and loggers (DecentLab GmbH, Dübendorf, Switzerland), have a low‐temperature sensitivity of < 0.3 µm °C^−1^, and data were not further corrected for temperature sensitivity. Continuously measured stem radius (SR) fluctuations were separated into growth‐induced irreversible stem expansion (GRO) and tree water deficit‐induced reversible stem shrinkage (TWD). TWD was determined according to the approach of Zweifel *et al*. (2016), assuming no cell growth during periods of stem shrinkage (zero growth concept). Thus, a period with TWD was defined as the time span when no net stem expansion occurs. Once the actual SR exceeds a previously detected SR maximum the TWD period ends and the differences (i.e. negative values) between the previous SR maximum and the SR readings during the TWD period (in 10 min resolution) were calculated, converted to positive values and defined as TWD (in µm). Thereafter, daily average TWD values were calculated.

### Root distribution

In July 2018, three soil cores were taken down to 50 cm soil depth, each close to one of the sample trees. We restricted sampling to that soil depth as a temporary waterlogged horizon occurred at *c*. 50 cm depth and beech roots are highly sensitive to anaerobic conditions (Kreuzwieser & Rennenberg, 2014) thus not penetrating into the saturated zone (Köstler *et al*., 1968). From these cores, the proportional root density (dry biomass of fine roots with diameter < 2 mm per soil volume; coarser roots are assumed not to be involved in water uptake) distribution over the profile was determined according to Volkmann *et al*. (2016a). The root distributions were assumed to be constant over the duration of the experiment.

### Isotope measurements

Soil and xylem water oxygen isotope composition (δ^18^O) was obtained nondestructively and continually by applying the setup detailed by Volkmann & Weiler (2014) and Volkmann *et al*. (2016a, 2016b,2016a, 2016b). In brief, water oxygen isotopologues from both the soil and the xylem (i.e. sapwood) were determined using the advection dilution sampling method (Volkmann & Weiler, 2014). The technique uses automatically controllable valve arrays to extract successively (compressed air) diluted water vapour from the soil air at various depths and from the tree sapwood via a probe network of small water‐repellent microporous tubes directly into a water isotope ratio infrared spectroscopy (IRIS) analyzer (L1102‐i WS‐CRDS; Picarro Inc., Santa Clara, CA, USA). Each probe was sampled for 10 min, and then sampling was switched automatically to the next one. The probes have a gas permeable head consisting of microporous hydrophobic polyethylene (Porex Technologies, Aachen, Germany) with 50 mm length and 10 mm outer diameter. The xylem probes were installed into a horizontal hole pre‐drilled into the main trunk sapwood of the beech trees at breast height and sealed with silicone to avoid exchange with ambient air. The soil probes were installed horizontally in both profiles (*c*. 5 m apart) at 5, 15, 30 and 45 cm depth in one of the profiles close to the ECH2O 5 TE sensors (see Table S1). None of the three trees was more than 5 m away from one of the two profiles. The probe design is described in detail by Volkmann & Weiler (2014) (soil) and Volkmann *et al*. (2016b) (xylem).

The obtained δ^18^O data of water vapour were normalized against the international reference Vienna Standard Mean Ocean Water (VSMOW) and are reported as δ^18^O (‰). Normalized liquid‐phase isotope signatures were obtained based on a specific on‐site calibration (see Volkmann & Weiler, 2014) every *c*. 2 h along with corrections for vapour concentration and temperature‐dependent isotopic liquid–vapour fractionation. For calibration, two probes were inserted into soil‐filled sealed containers. We used soil from the field site that first was oven‐dried and subsequently rewetted with water of specific, known oxygen isotope signatures (−4.4 and −19.5‰). The two standard containers were measured once per measurement cycle (consisting of the four depths in each soil profile and three trees). Thus one full measurement cycle lasted 130 min (10 min per probe × (3 tree probes + 8 soil probes + 2 standards)). Measurement precision (standard deviation of the standards) for δ^18^O was 0.3‰. To carry out corrections for water vapour concentration, we used a nonfractionating injection humidifier (Cellcraft F‐100; Cellcraft AB, Stockholm, Sweden) to create defined water vapour concentrations between 1000 and 30 000 ppm in a plexiglas mixing chamber and applied water of different known isotopic signatures to the water inlet of the humidifier. The humidified air was drawn into the IRIS and a δ^18^O reading was obtained. This procedure was performed in the laboratory before the field measurements. We plotted the δ^18^O (‰) readings against the water vapour concentrations (in ppm) and performed a fit with a logistic model providing *R* ^2^ values > 0.99 in repeated measurement sequences. The differences between the known and the modelled δ^18^O of water vapour were calculated and applied as water vapour concentration dependent correction factor. Within the δ^18^O range determined in this study no significant difference in the relationship between δ^18^O and water vapour concentration was found and thus all corrections applied were based on the correction factor for water vapour generated from water with a known oxygen isotopic signature of −8.2‰. Water vapour concentrations were always above 6000 ppm where the concentration dependent deviation becomes low and thus measurement precision is not compromised.

For comparison with traditional cryogenic water extraction and IRMS analysis, on 3 d during the experimental period, duplicate soil samples (5 (2.5–7.5), 15 (12.5–17.5), 30 (27.5–32.5), 45 (42.5–47.5) cm) were taken with soil corers close to the soil probes from both profiles. In addition, two sapwood cores per tree were collected on the same days. Soil and xylem water were extracted cryogenically (Treydte *et al*., 2014). In addition, precipitation (i.e. throughfall) in the forest stand was collected with three samplers made of a 2 l bottle with a funnel (diameter 30 cm) on top. Precipitation was collected from major rainfall events (> 1.5 mm) either directly after the event or the next day to avoid evaporative enrichment.

Analysis of δ^18^O in these water samples was performed with a thermal combustion/elemental analyzer coupled to a DELTA^PLUS^XP IRMS (temperature conversion elemental analyser‐IRMS; all Finnigan MAT, Bremen, Germany). Measurement precision was better than 0.3‰ (SD).

### Bayesian isotope mixing model and statistical analyses

Bayesian isotope mixing models (BIMMs) (Erhardt & Bedrick, 2013; Parnell *et al*., 2013) were applied to infer the spatial (i.e. depth) patterns of tree water uptake sources from daily averaged observed δ^18^O data (and SD) in the xylem and the four soil depths. A Bayesian inference approach was selected to estimate the mixture compositions in underdetermined systems, to account for uncertainty and variability in the isotopic data, and to generate potential solutions as true probability distributions. We applied the R‐package mixsiar (Stock *et al*., 2018). Relative root density distribution was included as an informative prior, and isotope values and concentration dependence (via soil water content in the different soil layers) were considered. The model run was performed with the Markov chain Monte Carlo run option ‘normal’ (chain length: 100 000; burn‐in: 50 000; thin: 50; number of chains: three) and residual * process error structure. Convergence of the models was checked using the Gelman–Rubin diagnostic. The posterior mean and SD vectors of proportional source contributions were estimated based on posterior probability densities from Gibbs sampler (Plummer, 2003; Stock *et al*., 2018) runs with each model. Posterior distributions were typically showing normality. The relative contribution of water uptake from the four different soil layers was multiplied by ground area‐based transpiration to obtain the absolute water extracted by the trees from each soil depth (in mm d^−1^).

Pearson’s correlation analysis was performed between either the relative or the absolute contributions of the different soil layers to tree water uptake and total (ground area‐based) transpiration or between daily (24 h) sums of transpiration (in l m^−2^ d^−1^) averaged over the three trees and daily (24 h) average VPD or VWC. Weekly values of the relative or absolute contribution of water uptake from a given soil layer (5 and 45 cm) were compared by repeated‐measurement ANOVA and Tukey *post hoc* tests. These analyses were performed with NCSS 2020 (NCCS, LLC, Kaysville, UT, USA).

## Results

### Method validation

**Fig. 1:**
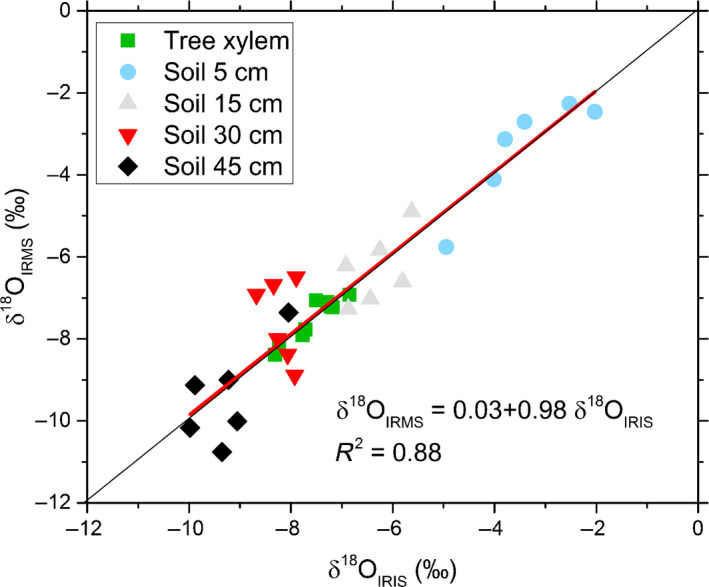
Relationship between the δ^18^O in soil and xylem of European beech from the *in situ* isotope ratio infrared spectroscopy (IRIS) ( δ^18^O_IRIS_) and from isotope ratio mass spectrometry (IRMS) (δ^18^O_IRMS_). For IRMS measurements, soil samples and trunk sapwood cores were cryogenically distilled to obtain the water, which was pyrolyzed and the produced carbon monoxide (CO) was directed into the IRMS. For each soil depths two cores were taken close to the *in situ* probes at three time points during the growing season (*n* = 6). At the same time points, sapwood cores were taken from the trunks of the three sample trees (*n* = 9). *In sit*u data from the time of sampling were taken for the regression analysis.

High agreement was found between the δ^18^O values from the *in situ* IRIS measurements and from samples taken close to the *in situ* probes from the soil and the tree stems and analysed with the classical technique (cryogenic distillation and temperature conversion elemental analyser‐IRMS measurement) (Fig. 1). The regression line was close to the 1 : 1 line, and there were no clear outliers, neither for the soil nor for xylem samples. These findings points to the fact that spectral interferences with plant‐produced volatile organic compounds (VOCs), which are known to affect δ^18^O measurements with IRIS (e.g. Volkmann *et al*., 2016b) did not play a role in the xylem water of beech trees. The average root mean square of residuals (*R*_s_) between the spectral model fits and the absorption spectra recorded by the IRIS instrument can be used as an indicator of the presence of organic contaminants (Schmidt *et al*., 2012) such as phenols and monoterpenes. Here, the average *R*_s_ of the *in situ* xylem water measurements over the whole measurement period was 0.6 and thus below the average of the regularly measured standards + 1 SD (0.67). According to Schmidt *et al*. (2012) this precludes major organic contamination and agrees with the close relationship between our IRIS and IRMS measurements. We thus applied no further post‐processing corrections (such as the ones, e.g. described by Martín Gómez *et al*. (2015)). For soil water, these results indicate that the water extracted by cryogenic distillation, which is assumed to include both mobile and tightly bound soil water, is not different in its δ^18^O as compared to the soil water that is in equilibrium with the water vapour trapped by the *in situ* IRIS system.

### Environmental conditions and tree responses

**Fig. 2:**
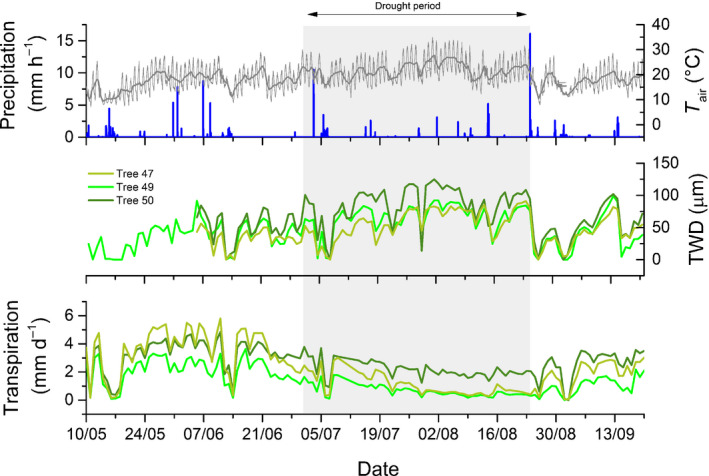
Precipitation, air temperature (*T*_air_), tree water deficit (TWD) and transpiration during summer 2018. Precipitation (blue bars) and *T*_air_ (grey lines) were measured outside the forest stand with a weather station being located in 200 m distance from the European beech trees. The thin grey line shows *T*_air_ in 10 min resolution whereas the bold line shows daily averages. Tree water deficit (derived from dendrometer measurements) and transpiration (calculated from sapflow) were determined in three European beech trees. The grey shaded area depicts the drought period. After the start of this period the soil volumetric water content in the upper soil layer stayed largely below 0.12 (see Fig. 3) and TWD started to increase. The end of the drought period was initiated by an intense rainfall event and indicated by the reduction in TWD and the increase in soil water content (see Fig. 3). Date format on the *x*‐axis: dd/mm.

**Fig. 3:**
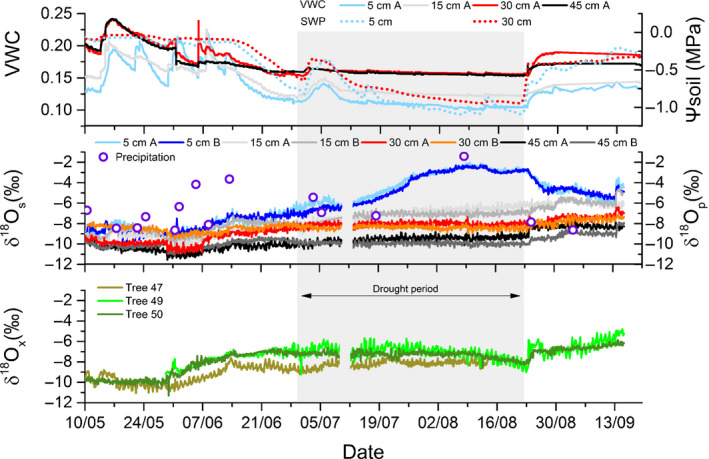
Volumetric water content (VWC), soil matric potential (Ψ_soil_) and δ^18^O in soil water (δ^18^O_s_), precipitation (δ^18^O_p_) and xylem water (δ^18^O_x_) during summer 2018. VWC and *in situ* δ^18^O_s_ (different coloured lines) were determined in 5, 15, 30 and 45 cm soil depths; Ψ_soil_ was measured in 5 and 30 cm. The δ^18^O_s_ measurements were performed in two soil profiles (A, B) and VWC in one of the profiles (A). The Ψ_soil_ was determined in another profile. The δ^18^O_p_ (open circles) was determined regularily after rainfall events. *In situ* probing for xylem water was carried out in the trunks of three European beech trees. The grey shaded area depicts the drought period. After the start of this period the soil volumetric water content in the upper soil layer stayed largely below 0.12 and tree water deficit (TWD) started to increase (see Fig. 2). No δ^18^O values were available from 10 July 2018 to 12 July 2018 due to a power failure. As of 14 August, only two trees were sampled due to the failure of a xylem probe. Date format on the *x*‐axis: dd/mm.

From mid‐May to mid‐June 2018, regular rainfall events summed to 153 mm at the study site (Fig. 2). After 13 June 2018, there was no rainfall for 2 wk, and rainfall events were sparse after that until 23 August 2018. The precipitation sums in July and August were 64 and 87 mm, respectively, and thus 50% and 26% lower compared to the long‐term average precipitation records (1980–2016) for the respective months (MeteoSwiss, Station Zurich‐Fluntern). The low precipitation in that period led to a reduction in VWCs, especially in 5 and 15 cm soil depth (Fig. 3). Comparably, a decrease in Ψ_soil_ in 5 and 30 cm depth was observed. After 29 June, Ψ_soil_ in the upper 5 cm reached values of −0.8 MPa (indicating soil drought conditions (Walthert *et al*., 2021)) while the TWD generally increased, interrupted by only a few and short TWD reductions due to small rainfall events (Fig. 2). On 29 June in one of the three trees (Tree 50, Fig. 2) TWD exceeded for the first time 100 µm. We thus defined the period between 29 June 2018 and 23 August 2018 as a drought period (indicated by the grey shaded areas in Figs 2, 3). Before the drought period, the average TWD was 39.2 ± 22.0 µm (maximum 91.7 µm) whereas during the drought period, it was 70.2 ± 27.2 µm (maximum 125.1 µm). There was also a clear increase in average air temperature from 17.1°C (maximum 28.9°C) before to 21.8°C (maximum 34.9°C) during the drought period. Transpiration started to drop already 2 wk before this drought period (Fig. 2) and amounted on average to 3.0 ± 1.4 mm d^−1^ before and to 1.3 ± 0.9 mm d^−1^ during the drought period. In the drought period, Ψ_soil_ decreased to below −0.9 MPa at 30 cm and −1 MPa at 5 cm (Fig. 3). During the drought period, daily transpiration sum was not significantly correlated to daily mean VPD (*r* = 0.14, *P* = 0.33) but to daily mean VWC (VWC at 5 cm soil depth: *r* = 0.65, *P* < 0.01).

On 23 August 2018, there was an intense rainfall event of 39 mm within 24 h, followed by a temperature drop (Fig. 2). As a consequence of this event and further subsequent precipitation, VWC increased in all four depths immediately as did Ψ_soil_ in the two depths measured (Fig. 3), and TWD dropped (Fig. 2), indicating the end of the drought period. Moreover, transpiration started to increase steadily and remained only low during rainfall events (Fig. 2). A correlation analysis showed that daily sums of transpiration in the period after drought were mainly related to daily mean VPD (*r* = 0.80, *P* < 0.01) and barely to soil water availability (daily mean VWC at 5 cm soil depth: *r* = 0.32, *P* = 0.18), meaning that transpiration was controlled mostly by atmospheric conditions rather than by soil moisture during this period.

### Isotopic patterns in soil and tree water pools

Soil water δ^18^O showed only a moderate depth gradient (with the highest enrichment in the shallowest layer) before the drought period. The gradient range between 5 and 45 cm soil depth was 2‰ on 10 May 2018 and increased to 3.4‰ on 29 June 2018 (start of the drought period). Thereafter the difference in soil water δ^18^O between 5 and 45 cm soil depth increased to up to 8‰ and only markedly decreased again after the drought‐ending precipitation event on 13 August 2018. The seasonal variation in soil water δ^18^O was lowest in the 30 and 45 cm soil layers where δ^18^O varied between −10.9‰ and −6.4‰ and −11.5‰ and −7.4‰, respectively. In the 5 cm soil layer, the high evaporative enrichment during the drought period led to a strong seasonal variation ranging from −10.9‰ to −2.0‰.

The δ^18^O of precipitation ranged between −8.7‰ and −1.4‰ and was mostly comparable to or more enriched than soil water from the upper 5 cm soil layer. There were three exceptions with δ^18^O values in precipitation lower compared to topsoil water, but two of them were related to events with low rainfall amounts (18 July; 3 September). The third was the intensive drought‐ending rainfall event that showed a δ^18^O of −7.9‰. As a consequence of this event, the δ^18^O of the soil water at 5 cm dropped by > 2‰ within a few days, whilst δ^18^O in the deeper soil layers increased, most likely due to the enriched shallow soil water being displaced into the deeper layers.

During the entire season, xylem water δ^18^O showed values between −11.3‰ and −4.8‰ with an increase by *c*. 2‰ from the end of May to the end of June, more or less constant values during the drought period and an increase by up to 2‰ within 24 h after the drought‐ending precipitation event. The three trees differed only slightly (maximum difference 2‰) and showed all comparable seasonal patterns. On 14 August, the xylem probe of Tree 47 failed, and thereafter data from only two trees were collected.

### Sources of tree water uptake

**Fig. 4:**
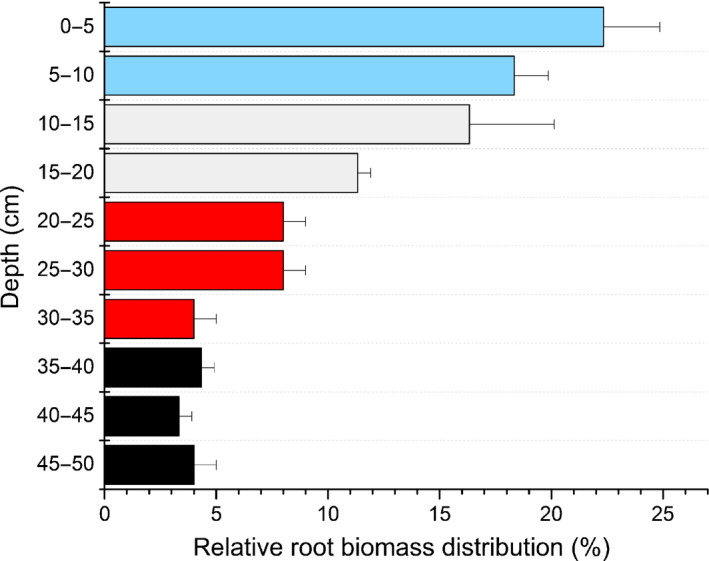
Relative fine root biomass distribution in beech. Fine roots were collected from three soil cores taken in proximity to the three European beech trees examined. Data shown are averages ± SD. The colour code groups the depths ranges of the root sampling to refer to the depths of the *in situ* isotope probing (Figs 3, 5).

Root distribution was used as informative prior for the BIMM; 41% of the fine roots were located in the upper 10 cm soil layer (Fig. 4). We assumed that roots from that depth range had access to the soil water probed from the uppermost soil layer (5 cm). Fine root mass decreased with depth, and in the soil layer below 35 cm there was only 12% of the total fine root biomass.

**Fig. 5:**
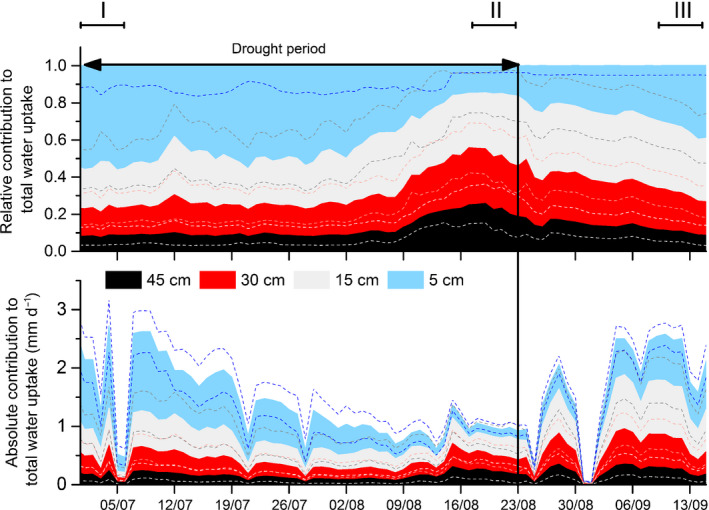
Relative (upper panel) and absolute (lower panel) contribution of soil water from different soil depths to total tree water uptake and use of European beech. The relative contribution of water from the different soil depths was calculated using a Bayesian isotope mixing model (BIMM) with daily resolution scaling for water content and using relative rooting distribution as informative prior. Data from 10 July 2018 to 12 July 2018 (lack of δ^18^O values) were linearly extrapolated. The relative depth distribution was multiplied with daily sums of transpiration (mm d^−1^) to obtain the absolute contribution. Data shown are mean values ± SE (dashed lines) (relative uptake 5 cm only −SE). Roman numerals indicate the periods for which the weekly values in Fig. 6 were calculated; I, beginning of drought; II, end of drought; III, after drought. Date format on the *x*‐axis: dd/mm.

**Fig. 6:**
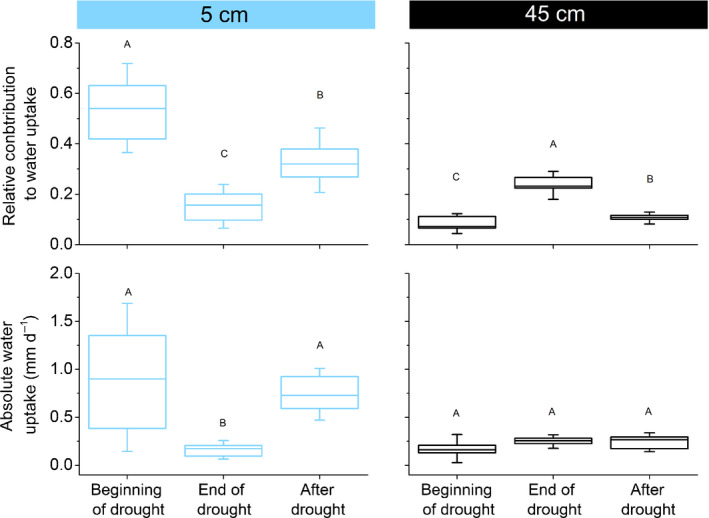
Boxplots for the weekly values of relative (upper panels) and absolute water uptake (lower panels) of European beech from the 5 and the 45 cm layer during the first (beginning of drought) and the last week (end of drought) of the drought period and in the last week of measurements (after drought). Letters A to C indicate significant differences (*P* < 0.05) between time periods as assessed by repeated measurement ANOVA and Tukey *post hoc* tests. Box width indicates the 25‰ and 75‰ and whiskers show SD. See Fig. 5 for the three periods the weekly values were calculated for.

The BIMM analyses showed that at the beginning (i.e. in the first week) of the drought period, 54 (± 18 SD)% of the water taken up by the trees originated from the 5 cm soil layer while 23 ± 2, 15 ± 5, and 8 ± 4% originated from the 15, 30, and 45 cm soil layers, respectively (Figs 5, 6). This distribution remained largely constant until the end of July, when the relative contribution of water from the uppermost soil layer started to decrease, while the share of the deeper soil layers increased. At the end of the drought period (i.e. in the week before the drought‐ending rainfall event), 16 ± 8% of the water uptake was sourced from the 5 cm soil layer, while the relative contributions of the other soil layers were 32 ± 8% (from 15 cm soil layer), 29 ± 11% (from 30 cm soil layer) and 23 ± 6% (from 45 cm soil layer). The decrease in the relative contribution of the upper soil layer with increasing drought duration was significant (Fig. 6) and correlated to tree water use (transpiration) (*r* = 0.44, *P* < 0.01; Table 1). The correlation analysis showed highly significant negative relationships between the relative contribution to water uptake from 5 cm and all other layers (Table 1). This points to the fact that with a reduced share of water from the uppermost soil layer, all other soil layers gained in relative importance (see Fig. 6 for 5 and 45 cm soil layers).

**Table 1:** Results of the correlation analyses for (a) the relative contributions of total water uptake from different soil depths over time and (b) absolute uptake of water either during drought or after drought.

Soil depths	5 cm	15 cm	30 cm	45 cm	Total transpiration
*(a) Relative contribution to total water uptake*					
During drought					
5 cm		−0.83***	−0.99***	−0.95***	0.44**
15 cm			0.75***	0.62***	−0.46**
30 cm				0.97***	−0.40**
45 cm					−0.39**
After drought					
5 cm		−0.30	−0.93***	−0.97***	0.32
15 cm			−0.04	0.14	0.21
30 cm				0.93***	−0.37
45 cm					−0.32
*(b) Total contribution to water uptake*					
During drought					
5 cm		0.81***	0.62***	0.23	0.94***
15 cm			0.92***	0.63***	0.95***
30 cm				0.87***	0.84***
45 cm					0.52***
After drought					
5 cm		0.90***	0.80***	0.66***	0.92***
15 cm			0.98***	0.91***	0.99***
30 cm				0.97***	0.97***
45 cm					0.90***

Results shown are Pearson’s correlation coefficients. ***, *P*‐value < 0.001; **, *P*‐value < 0.01. Negative significant relationships are indicated by red, positive significant relationships by blue colour.

After the drought ended, the relative contribution of water from the upper soil layer increased gradually, mainly at the expense of the two deepest layers (see Fig. 6 for 5 and 45 cm soil depths). This is also indicated by the highly significant negative correlations between time series of relative contribution of the topsoil layer to tree water uptake and those of 30 cm and 40 cm soil layers (5 cm vs 30 cm: *r* = −0.93; *P* < 0.001; 5 cm vs 45 cm: *r* = −0.97; *P* < 0.001; Table 1). In the last week of our measurements (8–15 September 2018) and thus *c*. 3 wk after the drought ended, the 5 cm soil layer again contributed to 34 ± 14% of the tree water use (Figs 5, 6) while the other soil layers contributed to 35 ± 12% (15 cm), 20 ± 9% (30 cm), and 11 ± 2% (45 cm).

While during the drought period, the relative water use from the 5 cm layer decreased and that from the deeper layer increased significantly (Fig. 6), total water use decreased (Figs 2, 5). As a consequence, while the absolute uptake from 5 cm decreased significantly (*P* < 0.05) from the first week (0.92 mm d^−1^) until the last week of drought (0.15 mm d^−1^) by 84% (Figs 5, 6), the amounts of water taken up from the other soil layers did not change significantly (see Fig. 6 for 45 cm). This indicates that deeper soil layers still constantly supplied water resources to trees but did not compensate for reduced water uptake from the topsoil during the drought period. This assumption is supported by the fact that mainly water uptake from the upper two soil layers (5 and 15 cm) was strongly correlated with total transpiration. By contrast, uptake from 45 cm showed only a moderate relationship with total transpiration during drought (*r* = 0.52; *P* < 0.001, Table 1), indicating that deep water uptake was less determined by total tree water use and thus seems to be a more or less constant reserve. This is also corroborated by the lack of a significant relationship in absolute water uptake during drought between the 5 cm and 45 cm soil layer (*r* = 0.23, Table 1).

After the drought, when there was plenty of water available in all soil layers and transpiration was controlled by VPD, the uptake from the different layers scaled with total water use (*r* > 0.9, *P* < 0.001, Table 1; Fig. 5). In the time period between 8 September 2018 and 15 September 2018 (after drought in Fig. 6), the absolute water uptake from the 5 cm topsoil layer was restored to levels at the start of the drought.

## Discussion

An *in situ* water isotopologue monitoring system allowed us to determine more than 14 600 δ^18^O values for soil water and xylem sap during one growing season. We used these data to characterize water uptake by soil depth for one of the economically and ecologically most important tree species in Central Europe. In the extremely hot and dry summer of 2018 (Schuldt *et al*., 2020), we captured the uptake dynamics as the topsoil dried and after drought was finally broken.

### The *in situ* IRIS approach achieves δ^18^O values comparable to cryogenic extraction and IRMS measurement

Organic compounds in soil and xylem water are known to interfere with the water isotopologue spectrum analysed by IRIS (Brand *et al*., 2009; West *et al*., 2010; Martín Gómez *et al*., 2015). Volkmann *et al*. (2016b) observed 4.3% lower xylem water δ^18^O values when using the same *in situ* IRIS technique as applied here compared to cryogenic extraction and IRMS analysis in *Acer campestre*. Based on the finding that the difference in δ^18^O between the two methods was positively correlated with *R*_s_, a measure of spectral noise, these authors speculated that plant‐produced VOCs such as methanol caused the bias. We did not observe such a method‐dependent δ^18^O difference in either soil or xylem water, which is in agreement with the mean samples *R*_s_ values being lower than that of standards +1 SD. Beech is known to produce monoterpenes (van Meeningen *et al*., 2016) but only low amounts of oxygenated volatile organic compounds (oVOCs; such as alcohols, ketones and aldehydes)(König *et al*., 1995). This lack of oVOC might explain the negligible organic interference observed in this study as especially alcohols are known to exert strong spectral effects (Brand *et al*., 2009; Martín Gómez *et al*., 2015; Millar *et al*., 2021).

Our method is fundamentally different from that of, for example West *et al*. (2010), who observed deviations of up to 15.4‰ between IRMS and IRIS measurements. Whereas they cryogenically extracted plant water before IRIS analysis, we relied on water vapour in equilibrium with xylem and soil water. Samples used for cryogenic extraction are normally frozen and freezing is known to releases cell contents, which are then co‐distilled with the water, leading to significant organic contamination (West *et al*., 2010). Moreover, the vacuum applied in cryogenic extraction might also promote the release of VOCs. In agreement with our results, Zhao *et al*. (2011) found only minor (−0.06‰) differences between IRMS and IRIS derived δ^18^O values in xylem water of *Populus euphratica*. With a borehole equilibrium technique where water vapour in a slow‐moving airstream guided through a stem borehole is allowed to get in isotopic equilibration with the xylem water and where the water vapour is then measured by IRIS, Marshall *et al*. (2020) observed no general deviation of xylem water δ^18^O from known source water δ^18^O. They thus concluded that organic contaminants do not play a role in such a flow‐through system, but warned that inaccurate measurements might be rather due to nonequilibrium conditions.

In conclusion, both species and soil type (see Martín Gómez *et al*., 2015) contribute to the specific volatile organic spectrum, but the type of probing also seems to play an important role for the reliability of IRIS measurements. Neither organic interferences nor nonequilibrium conditions seemed to impair our measurement system. Our results also showed no principle isotopic difference between cryogenically extracted bulk soil or xylem water and water vapour in equilibrium with soil or xylem water. This observation is in agreement with the work by Millar *et al*. (2018), who did not observe any deviation in δ^18^O in wheat stems between cryogenic extracted water and equilibrium water vapour. We conclude that with our *in situ* water isotopologue monitoring system either the same water pools are probed as the ones extracted by cryogenic distillation or that the soil water at our site and the stem water of European beech is completely mixed. We acknowledge that this might not necessarily be the case for clay dominated soils, where part of the water can be tightly bound and might not equilibrate easily. We can also assume that our *in situ* water isotopologue monitoring system might be more problematic with ring‐porous species with only a few cell rows of active xylem as the probes there might not only capture the water transported but also partially water stored in parts of the xylem do not contribute to axial water flow.

### Beech does not compensate reduced topsoil water availability with uptake from deeper soil layers

In 2018, large parts of Central Europe experienced a severe and long‐lasting summer drought with strong impacts on tree and forest functioning (Buras *et al*., 2020; Schuldt *et al*., 2020). European beech was especially affected, as indicated by extremely premature leaf senescence (Wohlgemuth *et al*., 2020). At our site, Ψ_soil_ values below −1 MPa were observed during the drought period indicating soil drought conditions at a level that can lead to xylem embolism in beech (Walthert *et al*., 2021). The fact that the trees suffered from severe drought stress was also indicated by the increased TWD during the drought period. TWD is largely determined by the moisture conditions in air (VPD) and soil (Ψ_soil_) (Zweifel *et al*., 2005) and is commonly used as a biological indicator of drought stress (Dietrich *et al*., 2018; Schäfer *et al*., 2019).

Beech is known to show a strong coupling of stomatal conductance and transpiration with VPD when soil water availability is high – as also seen in our study after drought release – but that relationship ceases under soil drought conditions (Granier *et al*., 2000; Gessler *et al*., 2004). We found that during the drought period, transpiration was (1) decoupled from VPD and mainly governed by VWC and (2) dropped by a factor of 2.3 compared to pre‐drought conditions, corroborating our conjecture of high drought stress for beech.

It is well known that trees from seasonally dry environments show high plasticity in the vertical distribution of water uptake (Dawson & Pate, 1996; David *et al*., 2013). Barbeta *et al*. (2015) observed in a Mediterranean ecosystem that the dominant species (*Quercus ilex*, *Arbutus unedo*, *Phillyrea latifolia*) had dimorphic root systems enabling them to access shallow soil water in the wet and cold season and to increase groundwater uptake in the dry summer. More recent results from temperate forest ecosystems also indicate plasticity in the proportional (i.e. relative) uptake from different soil layers (Brinkmann *et al*., 2019; Seeger & Weiler, 2021). Consequently, tree species with a deeper reaching rooting system such as *F. excelsior, F. sylvatica* and *A*. *pseudoplatanus* can source relatively more water from deeper soil layers during drought (Brinkmann *et al*., 2019). Also, Seeger & Weiler (2021), who used the same *in situ* IRIS system in a beech forest in the Swabian Jura growing on a rendzic Leptosol observed an increase in the relative contribution of water taken up from deeper soil layers (measured down to 60 cm) during a drought period. These findings agree with the results of our study as with the drying of the topsoil, an increasingly higher relative proportion of water was taken up from deeper soil layers (Fig. 5).

However, on a quantitative basis, there was no increase in the absolute amount of water taken up from the deepest soil layer during the course of the drought event (Fig. 6). Our results rather show that tree water use (expressed as transpiration) mainly depends on the water availability of the the upper soil layers. As opposed to other species such as oak (Volkmann *et al*., 2016a), beech trees at our site were unable to use additional deep soil water to compensate for reduced water uptake from the topsoil. The water‐pool in deeper soil layers is a relatively constant reserve, the exploitation of which is, however, not adjusted if the demand cannot be met from surface soils. As a consequence, we have to reject hypothesis 1, proposing a compensation effect of the deeper roots during drought stress.

We assume that the fine root depth profile with the highest root density in the uppermost soil layers and only a few fine roots deeper down is responsible for the observed vertical soil water use patterns leading to a restricted water uptake capacity in deeper layers. Increases in hydraulic conductivity of deep reaching roots mediated by aquaporin activity have been observed in other species to compensate for lower topsoil water availabity (Johnson *et al*., 2014). Such adjustment either did not occur in beech at our site or was insufficient to cause a detectable increase in water uptake from the deeper soil. Moreover, since soil drought strongly depresses tree belowground metabolic activity, carbon allocation to roots and root growth (Joseph *et al*., 2020; Leuschner, 2020) the ability of growing more roots in deeper layers during the drought seems restricted.

It might be speculated that the temporary waterlogging horizon present at our site prevented beech roots from exploring deeper soil layers under normal (nondrought) conditions as they are highly sensitive to anaerobic conditions (Kreuzwieser & Rennenberg, 2014). However, comparable rooting patterns for beech were observed in a dry beech stand on rendzic Leptosol (Gessler *et al*., 2005) with *c*. 80% of the fine roots in the upper 15 cm (58% in the present study). In general, the fine root abundance of beech shows an exponential decrease with depth and clearly highest fine root biomass in the upper 25 cm across different sites with different soil and climatic conditions (Büttner & Leuschner, 1994; Leuschner *et al*., 2004). We thus conclude that the inability of beech to compensate for low water availability in topsoils with water uptake from deeper soil layers, as observed in our study is not an exception and might occur on both moist and dry sites making beech highly susceptible to the expected impacts of increasing extreme events under global climate change.

### Water uptake from the topsoil recovers gradually after drought release and reaches pre‐drought values after a few weeks

After the drought‐ending rainfall event, both VWC and Ψ_soil_ increased rapidly within a few days and then levelled off but did not fully recover to the values observed in May before drought. The Ψ_soil_ values > −0.5 MPa reached *c*. 1 wk after the strong rainfall event and the air temperature drop on 23 August 2018 indicate that there was no drought stress any longer (Walthert *et al*., 2021). This assumption is corroborated by the fact that after the end of the drought event, transpiration was controlled by VPD and not by VWC signifying that tree water use was no longer primarily restricted by soil water availability. In contrast to the rapid increase in VWC in the upper 5 cm (see Fig. 3), the relative contribution of topsoil water to tree water use increased rather gradually over 3 wk (see Fig. 4). Thus, we conclude that the increase in water uptake from shallow soils, though starting immediately after the rainfall event, still lagged behind the rise in water availability. There might be two main reasons for such a lag: (1) Soil drought is known to decrease beech fine root abundance and growth rates and increase root mortality (Leuschner *et al*., 2001; Meier & Leuschner, 2008). Thus, the long‐lasting and strongly reduced water availability in the topsoil is likely to have led to a lower amount of living functional fine roots; the time it takes for regrowth of new roots might explain the observed lag. In support, Hagedorn *et al*. (2016) detected strong carbon allocation to beech roots after the release of a several months lasting drought, and this increase in carbon sink strength indicates increased carbon demand for root regrowth and repair. (2) Drought can reduce root hydraulic conductance either by anatomical changes of fine roots or by the downregulation of the root aquaporin expression (North & Nobel, 1992; Lo Gullo *et al*., 1998; Carmen Martínez‐Ballesta *et al*., 2003). The reduction of hydraulic conductance in fine roots from drought exposed shallow soil layers might prevent roots from loosing water taken up from the rooting system in deeper, moister soil layers. Volkmann *et al*. (2016a) observed a time lag between soil re‐wetting and root water uptake from shallow soils of several hours after a short 2‐wk drought period in oak seedlings. The authors attributed this very short delay to the time it takes to upregulate aquaporin expression and activity. It is known that fast recovery of water relations on the leaf level is also mediated by aquaporins (Perez‐Martin *et al*., 2014). In our case, the recovery of water uptake from the upper soils was slower and more gradual. Even though this does not exclude aquaporin involvement, we assume that root regrowth to compensate for lower fine root abundance or to replace roots with anatomically adjusted lower hydraulic conductance or xylem embolism might be the main mechanism to explain the observed time lag.

Still, in the time interval between 18 to 25 d (period III in Fig. 5; after drought in Fig. 6) after the end of the drought period, absolute water uptake in the 5 cm layer recovered to values at the onset of drought and thus we can accept hypothesis 2, which proposes a rapid recovery of water uptake from the topsoil once the water returns. According to the stress‐recovery concept proposed by Ruehr *et al*. (2019), a lagged but complete recovery after a stress event is indicative of only low amounts of structural damage and the need for tissue repair but not for tissue regrowth. For beech fine roots, however, increased growth after drought release might be additionally required to restore functioning due to their high susceptibility (e.g. cavitation or death) in response to water restriction (Leuschner, 2020).

### Conclusions

Our beech trees did not quantitatively compensate for restricted topsoil water availability by additional uptake from deeper soil layers. This inability helps to explain the widespread damage of beech during the extremely hot and dry summer of 2018 all over Europe (Schuldt *et al*., 2020; Wohlgemuth *et al*., 2020; Walthert *et al*., 2021) and is in agreement with the supposed drought susceptibility of beech (Rennenberg *et al*., 2004; Gessler *et al*., 2007). At our site, however, we also observed a rather fast restoration of water uptake, which also matches the high recovery potential of beech after drought (Rohner *et al*., 2021).

## Author contributions

AG, KT, AR, MW and SS planned and designed the research. AG, LB, KT, M Saurer, SS, CH, RZ, FH, M Schaub and KM conducted fieldwork and performed experiments. AG, LB, KM, MH, JM, ERF and FH analysed data. AG wrote the manuscript with input from all co‐authors.

## Supporting information


**Table S1** Measurements of oxygen isotope composition (δ^18^O) of soil water, volumetric soil water content (VWC) plus soil temperature (Soil T) and soil matric potential (Ψ_soil_) along soil depth.
Please note: Wiley Blackwell are not responsible for the content or functionality of any Supporting Information supplied by the authors. Any queries (other than missing material) should be directed to the *New Phytologist* Central Office.

## Data Availability

The data that support the findings of this study are available from the corresponding author upon request.

## References

[nph17767-bib-0001] Allen CD , Breshears DD , McDowell NG . 2015. On underestimation of global vulnerability to tree mortality and forest die‐off from hotter drought in the Anthropocene. Ecosphere 6: 129.

[nph17767-bib-0002] Allen CD , Macalady AK , Chenchouni H , Bachelet D , McDowell N , Vennetier M , Kitzberger T , Rigling A , Breshears DD , Hogg EH *et al*. 2010. A global overview of drought and heat‐induced tree mortality reveals emerging climate change risks for forests. Forest Ecology and Management 259: 660–684.

[nph17767-bib-0003] Allen MF . 2007. Mycorrhizal fungi: highways for water and nutrients in arid soils. Vadose Zone Journal 6: 291–297.

[nph17767-bib-0004] Barbeta A , Mejía‐Chang M , Ogaya R , Voltas J , Dawson TE , Peñuelas J . 2015. The combined effects of a long‐term experimental drought and an extreme drought on the use of plant–water sources in a Mediterranean forest. Global Change Biology 21: 1213–1225. 25359123 10.1111/gcb.12785

[nph17767-bib-0005] Barbeta A , Peñuelas J . 2017. Relative contribution of groundwater to plant transpiration estimated with stable isotopes. Scientific Reports 7: 10580. 28874685 10.1038/s41598-017-09643-x PMC5585407

[nph17767-bib-0006] Bello J , Hasselquist NJ , Vallet P , Kahmen A , Perot T , Korboulewsky N . 2019. Complementary water uptake depth of *Quercus petraea* and *Pinus sylvestris* in mixed stands during an extreme drought. Plant and Soil 437: 93–115.

[nph17767-bib-0007] Bowling DR , Schulze ES , Hall SJ . 2017. Revisiting streamside trees that do not use stream water: can the two water worlds hypothesis and snowpack isotopic effects explain a missing water source? Ecohydrology 10: e1771.

[nph17767-bib-0008] Brand WA , Geilmann H , Crosson ER , Rella CW . 2009. Cavity ring‐down spectroscopy versus high‐temperature conversion isotope ratio mass spectrometry; a case study on δ^2^H and δ^18^O of pure water samples and alcohol/water mixtures. Rapid Communications in Mass Spectrometry 23: 1879–1884. 19449320 10.1002/rcm.4083

[nph17767-bib-0009] Brandes E , Wenninger J , Koeniger P , Schindler D , Rennenberg H , Leibundgut C , Mayer H , Gessler A . 2007. Assessing environmental and physiological controls over water relations in a Scots pine (*Pinus sylvestris* L.) stand through analyses of stable isotope composition of water and organic matter. Plant, Cell & Environment 30: 113–127. 10.1111/j.1365-3040.2006.01609.x 17177880

[nph17767-bib-0010] Brinkmann N , Eugster W , Buchmann N , Kahmen A . 2019. Species‐specific differences in water uptake depth of mature temperate trees vary with water availability in the soil. Plant Biology 21: 71–81. 30184305 10.1111/plb.12907

[nph17767-bib-0011] Brooks JR , Barnard HR , Coulombe R , McDonnell JJ . 2010. Ecohydrologic separation of water between trees and streams in a Mediterranean climate. Nature Geoscience 3: 100–104.

[nph17767-bib-0012] Buras A , Rammig A , Zang CS . 2020. Quantifying impacts of the 2018 drought on European ecosystems in comparison to 2003. Biogeosciences 17: 1655–1672.

[nph17767-bib-0013] Büttner V , Leuschner C . 1994. Spatial and temporal patterns of fine‐root abundance in a mixed Oak Beech Forest. Forest Ecology and Management 70: 11–21.

[nph17767-bib-0014] Carmen Martínez‐Ballesta M , Aparicio F , Pallás V , Martínez V , Carvajal M . 2003. Influence of saline stress on root hydraulic conductance and PIP expression in *Arabidopsis* . Journal of Plant Physiology 160: 689–697. 12872491 10.1078/0176-1617-00861

[nph17767-bib-0015] Chen Y , Helliker BR , Tang X , Li F , Zhou Y , Song X . 2020. Stem water cryogenic extraction biases estimation in deuterium isotope composition of plant source water. Proceedings of the National Academy of Sciences, USA 117: 33345–33350. 10.1073/pnas.2014422117 PMC7776815 33318208

[nph17767-bib-0016] Churkina G , Running SW . 1998. Contrasting climatic controls on the estimated productivity of global terrestrial biomes. Ecosystems 1: 206–215.

[nph17767-bib-0017] Ciais P , Reichstein M , Viovy N , Granier A , Ogee J , Allard V , Aubinet M , Buchmann N , Bernhofer C , Carrara A *et al*. 2005. Europe‐wide reduction in primary productivity caused by the heat and drought in 2003. Nature 437: 529–533. 16177786 10.1038/nature03972

[nph17767-bib-0018] Costelloe JF , Payne E , Woodrow IE , Irvine EC , Western AW , Leaney FW . 2008. Water sources accessed by arid zone riparian trees in highly saline environments, Australia. Oecologia 156: 43–52. 18270743 10.1007/s00442-008-0975-4

[nph17767-bib-0019] David TS , Pinto CA , Nadezhdina N , Kurz‐Besson C , Henriques MO , Quilhó T , Cermak J , Chaves MM , Pereira JS , David JS . 2013. Root functioning, tree water use and hydraulic redistribution in *Quercus* *suber* trees: a modeling approach based on root sap flow. Forest Ecology and Management 307: 136–146.

[nph17767-bib-0020] Dawson TE , Ehleringer JR . 1991. Streamside trees that do not use stream water. Nature 350: 335–337.

[nph17767-bib-0021] Dawson TE , Pate JS . 1996. Seasonal water uptake and movement in root systems of Australian phraeatophytic plants of dimorphic root morphology: a stable isotope investigation. Oecologia 107: 13–20. 28307187 10.1007/BF00582230

[nph17767-bib-0022] Dietrich L , Zweifel R , Kahmen A . 2018. Daily stem diameter variations can predict the canopy water status of mature temperate trees. Tree Physiology 38: 941–952. 29554370 10.1093/treephys/tpy023

[nph17767-bib-0023] Ehleringer JR , Roden JS , Dawson T . 2000. Assessing ecosystem‐level water relations through stable isotope ratio analyses. In: Sala OE , Jackson R , Mooney HA , Howarth R , eds. Methods in ecosystem science. New York, NY, USA: Springer Verlag, 181–198.

[nph17767-bib-0024] Erhardt EB , Bedrick EJ . 2013. A Bayesian framework for stable isotope mixing models. Environmental and Ecological Statistics 20: 377–397.

[nph17767-bib-0025] Gangi L , Rothfuss Y , Ogée J , Wingate L , Vereecken H , Brüggemann N . 2015. A new method for *in situ* measurements of oxygen isotopologues of soil water and carbon dioxide with high time resolution. Vadose Zone Journal 14: vzj2014.2011.0169.

[nph17767-bib-0026] Gessler A , Jung K , Gasche R , Papen H , Heidenfelder A , Borner E , Metzler B , Augustin S , Hildebrand E , Rennenberg H . 2005. Climate and forest management influence nitrogen balance of European beech forests: microbial N transformations and inorganic N net uptake capacity of mycorrhizal roots. European Journal of Forest Research 124: 95–111.

[nph17767-bib-0027] Gessler A , Keitel C , Kreuzwieser J , Matyssek R , Seiler W , Rennenberg H . 2007. Potential risks for European beech (*Fagus sylvatica* L.) in a changing climate. Trees‐Structure and Function 21: 1–11.

[nph17767-bib-0028] Gessler A , Keitel C , Nahm M , Rennenberg H . 2004. Water shortage affects the water and nitrogen balance in central European beech forests. Plant Biology 6: 289–298. 15143437 10.1055/s-2004-820878

[nph17767-bib-0029] Granier A , Biron P , Köstner B , Gay L , Najjar G . 1996. Comparisons of xylem sap flow and water vapour flux at the stand level and derivation of canopy conductance for Scots pine. Theoretical and Applied Climatology 53: 115–122.

[nph17767-bib-0030] Granier A , Biron P , Lemoine D . 2000. Water balance, transpiration and canopy conductance in two beech stands. Agricultural and Forest Meteorology 100: 291–308.

[nph17767-bib-0031] Hagedorn F , Joseph J , Peter M , Luster J , Pritsch K , Geppert U , Kerner R , Molinier V , Egli S , Schaub M *et al*. 2016. Recovery of trees from drought depends on belowground sink control. Nature Plants 2: 16111. 27428669 10.1038/nplants.2016.111

[nph17767-bib-0032] Hanewinkel M , Cullmann DA , Schelhaas MJ , Nabuurs GJ , Zimmermann NE . 2013. Climate change may cause severe loss in the economic value of European forest land. Nature Climate Change 3: 203–207.

[nph17767-bib-0033] Johnson DM , Sherrard ME , Domec J‐C , Jackson RB . 2014. Role of aquaporin activity in regulating deep and shallow root hydraulic conductance during extreme drought. Trees 28: 1323–1331.

[nph17767-bib-0034] Jones HG . 1992. Plants and microclimate: a quantitative approach to environmental plant physiology. Cambridge, UK: Cambridge University Press.

[nph17767-bib-0035] Joseph J , Gao D , Backes B , Bloch C , Brunner I , Gleixner G , Haeni M , Hartmann H , Hoch G , Hug C *et al*. 2020. Rhizosphere activity in an old‐growth forest reacts rapidly to changes in soil moisture and shapes whole‐tree carbon allocation. Proceedings of the National Academy of Sciences, USA 117: 24885–24892. 10.1073/pnas.2014084117 PMC7547207 32958662

[nph17767-bib-0036] Keitel C , Adams MA , Holst T , Matzarakis A , Mayer H , Rennenberg H , Gessler A . 2003. Carbon and oxygen isotope composition of organic compounds in the phloem sap provides a short‐term measure for stomatal conductance of European beech (*Fagus sylvatica* L.). Plant, Cell & Environment 26: 1157–1168.

[nph17767-bib-0037] König G , Brunda M , Puxbaum H , Hewitt CN , Duckham SC , Rudolph J . 1995. Relative contribution of oxygenated hydrocarbons to the total biogenic VOC emissions of selected mid‐European agricultural and natural plant species. Atmospheric Environment 29: 861–874.

[nph17767-bib-0038] Köstler J , Brückner E , Bibelriether H . 1968. Die Wurzeln der Waldbäume. Hamburg, Germany: Oaul Parey.

[nph17767-bib-0039] Kreuzwieser J , Rennenberg H . 2014. Molecular and physiological responses of trees to waterlogging stress. Plant, Cell & Environment 37: 2245–2259. 10.1111/pce.12310 24611781

[nph17767-bib-0040] Lanning M , Wang L , Benson M , Zhang Q , Novick KA . 2020. Canopy isotopic investigation reveals different water uptake dynamics of maples and oaks. Phytochemistry 175: 112389. 32330693 10.1016/j.phytochem.2020.112389

[nph17767-bib-0041] Leuschner C . 2020. Drought response of European beech (*Fagus sylvatica* L.) – a review. Perspectives in Plant Ecology, Evolution and Systematics 47: 125576.

[nph17767-bib-0042] Leuschner C , Backes K , Hertel D , Schipka F , Schmitt U , Terborg O , Runge M . 2001. Drought responses at leaf, stem and fine root levels of competitive *Fagus sylvatica* L. and *Quercus petraea* (Matt.) Liebl. trees in dry and wet years. Forest Ecology and Management 149: 33–46.

[nph17767-bib-0043] Leuschner C , Hertel D , Schmid I , Koch O , Muhs A , Hölscher D . 2004. Stand fine root biomass and fine root morphology in old‐growth beech forests as a function of precipitation and soil fertility. Plant and Soil 258: 43–56.

[nph17767-bib-0044] Lo Gullo MA , Nardini A , Salleo S , Tyree MT . 1998. Changes in root hydraulic conductance (*K* _R_) of *Olea oleaster* seedlings following drought stress and irrigation. New Phytologist 140: 25–31.

[nph17767-bib-0045] Lüttschwager D , Jochheim H . 2020. Drought primarily reduces canopy transpiration of exposed beech trees and decreases the share of water uptake from deeper soil layers. Forests 11: 537.

[nph17767-bib-0046] Marshall JD , Cuntz M , Beyer M , Dubbert M , Kuehnhammer K . 2020. Borehole equilibration: testing a new method to monitor the isotopic composition of tree xylem water *in situ* . Frontiers in Plant Science 11: 358. 32351515 10.3389/fpls.2020.00358 PMC7175398

[nph17767-bib-0047] Martín Gómez P , Barbeta A , Voltas J , Penuelas J , Dennis K , Palacio S , Dawson TE , Ferrio JP . 2015. Isotope‐ratio infrared spectroscopy: a reliable tool for the investigation of plant–water sources? New Phytologist 207: 914–927. 25790288 10.1111/nph.13376

[nph17767-bib-0048] McDonnell JJ . 2014. The two water worlds hypothesis: ecohydrological separation of water between streams and trees? Wires Water 1: 323–329.

[nph17767-bib-0049] van Meeningen Y , Schurgers G , Rinnan R , Holst T . 2016. BVOC emissions from English oak (*Quercus* *robur*) and European beech (*Fagus sylvatica*) along a latitudinal gradient. Biogeosciences 13: 6067–6080.

[nph17767-bib-0050] Meier IC , Leuschner C . 2008. Genotypic variation and phenotypic plasticity in the drought response of fine roots of European beech. Tree Physiology 28: 297–309. 18055440 10.1093/treephys/28.2.297

[nph17767-bib-0051] Millar C , Janzen K , Nehemy MF , Koehler G , Hervé‐Fernández P , McDonnell JJ . 2021. Organic contamination detection for isotopic analysis of water by laser spectroscopy. Rapid Communications in Mass Spectrometry 35: e9118. 33939862 10.1002/rcm.9118

[nph17767-bib-0052] Millar C , Pratt D , Schneider DJ , McDonnell JJ . 2018. A comparison of extraction systems for plant water stable isotope analysis. Rapid Communications in Mass Spectrometry 32: 1031–1044. 29645300 10.1002/rcm.8136

[nph17767-bib-0053] North GB , Nobel PS . 1992. Drought‐induced changes in hydraulic conductivity and structure in roots of *Ferocactus* *acanthodes* and *Opuntia* *ficus‐indica* . New Phytologist 120: 9–19.

[nph17767-bib-0054] Parnell AC , Phillips DL , Bearhop S , Semmens BX , Ward EJ , Moore JW , Jackson AL , Grey J , Kelly DJ , Inger R . 2013. Bayesian stable isotope mixing models. Environmetrics 24: 387–399.

[nph17767-bib-0055] Perez‐Martin A , Michelazzo C , Torres‐Ruiz JM , Flexas J , Fernández JE , Sebastiani L , Diaz‐Espejo A . 2014. Regulation of photosynthesis and stomatal and mesophyll conductance under water stress and recovery in olive trees: correlation with gene expression of carbonic anhydrase and aquaporins. Journal of Experimental Botany 65: 3143–3156. 24799563 10.1093/jxb/eru160 PMC4071832

[nph17767-bib-0056] Plamboeck AH , Grip H , Nygren U . 1999. A hydrological tracer study of water uptake depth in a Scots pine forest under two different water regimes. Oecologia 119: 452–460. 28307769 10.1007/s004420050807

[nph17767-bib-0057] Plummer M . 2003. JAGS: A program for analysis of Bayesian graphical models using Gibbs sampling. In: Hornik K , Leisch F , Zeileis A , eds. Proceedings of the 3rd international workshop on distributed statistical computing. Vienna, Austria. [WWW document] URL https://www.r‐project.org/conferences/DSC‐2003/Proceedings/Plummer.pdf.

[nph17767-bib-0058] Poorter H , Niklas KJ , Reich PB , Oleksyn J , Poot P , Mommer L . 2012. Biomass allocation to leaves, stems and roots: meta‐analyses of interspecific variation and environmental control. New Phytologist 193: 30–50. 22085245 10.1111/j.1469-8137.2011.03952.x

[nph17767-bib-0059] Porporato A , Daly E , Rodriguez‐Iturbe I . 2004. Soil water balance and ecosystem response to climate change. The American Naturalist 164: 625–632. 10.1086/424970 15540152

[nph17767-bib-0060] Rennenberg H , Seiler W , Matyssek R , Gessler A , Kreuzwieser J . 2004. Die Buche (*Fagus sylvatica* L.)–ein Waldbaum ohne Zukunft im südlichen Mitteleuropa. Allgemeine Forst und Jagdzeitung 175: 210–224.

[nph17767-bib-0061] Rohner B , Kumar S , Liechti K , Gessler A , Ferretti M . 2021. Tree vitality indicators revealed a rapid response of beech forests to the 2018 drought. Ecological Indicators 120: 106903.

[nph17767-bib-0062] Ruehr NK , Grote R , Mayr S , Arneth A . 2019. Beyond the extreme: recovery of carbon and water relations in woody plants following heat and drought stress. Tree Physiology 39: 1285–1299. 30924906 10.1093/treephys/tpz032 PMC6703153

[nph17767-bib-0063] Schäfer C , Rötzer T , Thurm EA , Biber P , Kallenbach C , Pretzsch H . 2019. Growth and tree water deficit of mixed norway spruce and European beech at different heights in a tree and under heavy drought. Forests 10: 577.

[nph17767-bib-0064] Schmidt M , Maseyk K , Lett C , Biron P , Richard P , Bariac T , Seibt U . 2012. Reducing and correcting for contamination of ecosystem water stable isotopes measured by isotope ratio infrared spectroscopy. Rapid Communications in Mass Spectrometry 26: 141–153. 22173802 10.1002/rcm.5317

[nph17767-bib-0065] Schuldt B , Buras A , Arend M , Vitasse Y , Beierkuhnlein C , Damm A , Gharun M , Grams TEE , Hauck M , Hajek P *et al*. 2020. A first assessment of the impact of the extreme 2018 summer drought on Central European forests. Basic and Applied Ecology 45: 86–103.

[nph17767-bib-0066] Seeger S , Weiler M . 2021. Temporal dynamics of tree xylem water isotopes: in‐situ monitoring and modelling. Biogeosciences Discussions 2021: 1–41.

[nph17767-bib-0067] Sprenger M , Leistert H , Gimbel K , Weiler M . 2016. Illuminating hydrological processes at the soil–vegetation–atmosphere interface with water stable isotopes. Reviews of Geophysics 54: 674–704.

[nph17767-bib-0068] Stahl C , Hérault B , Rossi V , Burban B , Bréchet C , Bonal D . 2013. Depth of soil water uptake by tropical rainforest trees during dry periods: does tree dimension matter? Oecologia 173: 1191–1201. 23852028 10.1007/s00442-013-2724-6

[nph17767-bib-0069] Stock BC , Jackson AL , Ward EJ , Parnell AC , Phillips DL , Semmens BX . 2018. Analyzing mixing systems using a new generation of Bayesian tracer mixing models. PeerJ 6: e5096. 29942712 10.7717/peerj.5096 PMC6015753

[nph17767-bib-0070] Treydte K , Boda S , Graf Pannatier E , Fonti P , Frank D , Ullrich B , Saurer M , Siegwolf R , Battipaglia G , Werner W *et al*. 2014. Seasonal transfer of oxygen isotopes from precipitation and soil to the tree ring: source water versus needle water enrichment. New Phytologist 202: 772–783. 24602089 10.1111/nph.12741

[nph17767-bib-0071] Volkmann THM , Haberer K , Gessler A , Weiler M . 2016a. High‐resolution isotope measurements resolve rapid ecohydrological dynamics at the soil–plant interface. New Phytologist 210: 839–849. 26864434 10.1111/nph.13868

[nph17767-bib-0072] Volkmann THM , Kühnhammer K , Herbstritt B , Gessler A , Weiler M . 2016b. A method for *in situ* monitoring of the isotope composition of tree xylem water using laser spectroscopy. Plant, Cell & Environment 9: 2055–2063. 10.1111/pce.12725 27260852

[nph17767-bib-0073] Volkmann THM , Weiler M . 2014. Continual *in situ* monitoring of pore water stable isotopes in the subsurface. Hydrology and Earth System Sciences 18: 1819–1833.

[nph17767-bib-0074] Walthert L , Ganthaler A , Mayr S , Saurer M , Waldner P , Walser M , Zweifel R , von Arx G . 2021. From the comfort zone to crown dieback: sequence of physiological stress thresholds in mature European beech trees across progressive drought. Science of the Total Environment 753: 141792. 33207466 10.1016/j.scitotenv.2020.141792

[nph17767-bib-0075] Walthert L , Schleppi P . 2018. Equations to compensate for the temperature effect on readings from dielectric Decagon MPS‐2 and MPS‐6 water potential sensors in soils. Journal of Plant Nutrition and Soil Science 181: 749–759.

[nph17767-bib-0076] West AG , Goldsmith GR , Brooks PD , Dawson TE . 2010. Discrepancies between isotope ratio infrared spectroscopy and isotope ratio mass spectrometry for the stable isotope analysis of plant and soil waters. Rapid Communications in Mass Spectrometry 24: 1948–1954. 20552579 10.1002/rcm.4597

[nph17767-bib-0077] Wohlgemuth T , Kistler M , Aymon C , Hagedorn F , Gessler A , Gossner MM , Queloz V , Vögtli I , Wasem U , Vitasse Y *et al*. 2020. Früher Laubfall der Buche während der Sommertrockenheit 2018: resistenz oder Schwächesymptom? Schweizerische Zeitschrift fur Forstwesen 171: 257–269.

[nph17767-bib-0078] Yang B , Wen X , Sun X . 2015. Seasonal variations in depth of water uptake for a subtropical coniferous plantation subjected to drought in an East Asian monsoon region. Agricultural and Forest Meteorology 201: 218–228.

[nph17767-bib-0079] Zhao L , Xiao H , Zhou J , Wang L , Cheng G , Zhou M , Yin L , McCabe MF . 2011. Detailed assessment of isotope ratio infrared spectroscopy and isotope ratio mass spectrometry for the stable isotope analysis of plant and soil waters. Rapid Communications in Mass Spectrometry 25: 3071–3082. 21953962 10.1002/rcm.5204

[nph17767-bib-0080] Zweifel R , Haeni M , Buchmann N , Eugster W . 2016. Are trees able to grow in periods of stem shrinkage? New Phytologist 211: 839–849. 27189708 10.1111/nph.13995

[nph17767-bib-0081] Zweifel R , Zimmermann L , Newbery DM . 2005. Modeling tree water deficit from microclimate: an approach to quantifying drought stress. Tree Physiology 25: 147–156. 15574396 10.1093/treephys/25.2.147

