# Supplementary material for: Drought reduces water uptake in beech from the drying topsoil, but no compensatory uptake occurs from deeper soil layers
Source: New Phytol. 2021 Oct 15;233(1):194–206. doi: 10.1111/nph.17767 (PMC9293437; doi:10.1111/nph.17767)
Supplement: Supplementary file 1 — Table S1 Measurements of oxygen isotope composition (δ18O) of soil water, volumetric soil water content (VWC) plus soil temperature (Soil T) and soil matric potential (Ψsoil) along soil depth. Please note: Wiley Blackwell are not responsible for the content or functionality of any Supporting Information supplied by the authors. Any queries (other than missing material) should be directed to the New Phytologist Central Office. [file NPH-233-194-s001.pdf]

New Phytologist Supporting Information

Drought reduces water uptake in beech from the drying topsoil, but no compensatory uptake occurs from deeper soil layers

Arthur Gessler, Lukas Bächli, Elham Rouholahnejad Freund, Kerstin Treydte, Marcus Schaub, Matthias Haeni, Markus Weiler, Stefan Seeger, John Marshall, Christian Hug, Roman Zweifel, Frank Hagedorn, Andreas Rigling, Matthias Saurer, Katrin Meusburger

Article acceptance date: 26 September 2021

Soil depth (cm)	Soil profile A		Soil profile B	Soil profile C
5	$\delta^{18}O$	VWC/Soil T	$\delta^{18}O$	Ψ_{soil}
15	$\delta^{18}O$	VWC/Soil T	$\delta^{18}O$	
30	$\delta^{18}O$	VWC/Soil T	$\delta^{18}O$	Ψ_{soil}
45	$\delta^{18}O$	VWC/Soil T	$\delta^{18}O$	

Table S1: Measurements of oxygen isotope composition ($\delta^{18}O$) of soil water, volumetric soil water content (VWC) plus soil temperature (Soil T) and soil matric potential (Ψ_{soil}) along soil depth. $\delta^{18}O$ was measured in two profiles and VWC/Soil T in one of these two. Ψ_{soil} was measured in an additional profile
